# *Candida* Bloodstream Infections in Italy: Changing Epidemiology during 16 Years of Surveillance

**DOI:** 10.1155/2015/256580

**Published:** 2015-05-07

**Authors:** Giuseppina Caggiano, Caterina Coretti, Nicola Bartolomeo, Grazia Lovero, Osvalda De Giglio, Maria Teresa Montagna

**Affiliations:** Hygiene Section, Department of Biomedical Science and Human Oncology, University of Bari, Piazza G. Cesare 11, 70124 Bari, Italy

## Abstract

Although considerable progress has been made in the management of patients with invasive fungal infections, *Candida* bloodstream infections are still widespread in hospital settings. Incidence rates vary geographically, often because of different patient populations. The aim of the present study was to describe the epidemiology of candidemia, to analyze the trend of species distribution, and to measure the *in vitro* susceptibility to antifungal drugs in a university Italian hospital from 1998 to 2013. The antifungal susceptibility for all *Candida* isolates was evaluated by broth microdilution assay (CLSI M27-A3 document). Of 394 episodes of candidemia, the average incidence was 3.06/10 000 admissions. *C. albicans* and non-*albicans Candida* species caused 44.2% and 55.8% of the episodes, respectively. *C. parapsilosis* (62.2%) was the most common non-*albicans.* *C. albicans* predominated in almost all departments whereas *C. parapsilosis* was found in adult and paediatric oncohaematology units (34.8% and 77.6%, resp.). Overall, mortality occurred in 111 (28.2%) patients. Death occurred most often in intensive care units (47.1%) and specialist surgeries (43.7%). Most of the isolates were susceptible to antifungal drugs, but there was an upward trend for azole (*P* < 0.05). In conclusion, this study emphasizes the importance of monitoring local epidemiologic data and the diversity of patient groups affected.

## 1. Introduction

For over 20 years,* Candida* bloodstream infections (BSIs) have been increasing significantly worldwide, representing an important infective complication in hospitalized patients with medical and surgical disorders [1].* Candida* spp. are the fourth leading cause of nosocomial BSIs in the USA and the sixth in Europe [2–4]. Though the* Candida* species distribution varies, there is a well-documented diffusion of* Candida albicans* across geographical regions [5]. The epidemiology of candidemia generally appears complex and is characterized by a substantial regional and temporal variability. In fact, different incidence rates and new emerging species vary geographically in frequency of isolation, often because of different patient populations or study methodologies [6–8]. Despite advances in medical interventions, the development of novel antifungal drugs, and the infrequent resistance to common antifungal agents, mortality rates are still unacceptably high, ranging from 29% to 76%, with an attributable mortality of 49% [1]. This issue is largely due to factors such as the lack of specific clinical findings, the relatively slow and insensitive diagnostic tests that complicate the early recognition of fungal infections, and delays in the administration of appropriate therapy. The aim of this study was to describe the epidemiology of* Candida* BSIs in a hospital in Southern Italy during 16 years of observation and to analyze the trend in species distribution and* in vitro* susceptibility to common antifungal drugs.

## 2. Patients and Methods

### 2.1. Study Design

This observational study was carried out from January 1998 to December 2013 in a university hospital in Apulia, Southern Italy. The hospital is a 1400-bed teaching hospital that attends to cases of high complexity in medicine and surgery, including solid organ and haematopoietic stem cell transplants. All patients with BSI caused by* Candida* spp., when isolates were available, were included. An episode of candidemia was defined as* Candida* infection involving at least one blood culture.

For each patient, only the first episode of* Candida* BSI was recorded. Events were considered separate if they occurred at least 30 days apart or were caused by different species. Patients with candidemia were followed prospectively for 30 days after diagnosis.

Neither ethical approval nor patient consensus was considered necessary because we did not use patient accessible data or additional samples other than those obtained during the routine activity of laboratories. All data were collected using standardized case report forms.

### 2.2. Laboratory Procedures

Blood cultures were made using a lysis-centrifugation system (Isolator, DuPont Co., Wilmington, DE, USA). The samples were cultured on two plates of Sabouraud dextrose agar with 0.05% chloramphenicol (Bio-Rad, Marnes-la-Coquette, France), incubated at 36°C (±1) and 28°C (±1), and examined daily for 10 days. The yeast isolates were identified by analysis of biochemical patterns by ID32C kit (bioMèrieux, Marcy l'Étoile, France) and were frozen at −70°C until further investigation.

The antifungal susceptibility tests were performed for all* Candida* spp. isolates. The following drugs were supplied by the manufacturers as pure standard compounds: anidulafungin (AND), fluconazole (FLC), voriconazole (VRC) (Pfizer Pharmaceuticals, Groton, CT, USA), caspofungin (CSP) (Merck & Co, Inc., Whitehouse Station, NJ, USA), posaconazole (PSC) (Schering-Plough Corporation, Kenilworth, NJ, USA), micafungin (MCF) (Astellas Pharma, Tokyo, Japan), and amphotericin B (AmB) obtained from Sigma (Sigma-Aldrich, Milan, Italy). Antifungal susceptibility was evaluated by a broth microdilution assay performed according to the Clinical and Laboratory Standards Institute recommendations (CLSI M27-A3 document) [13]. Before the beginning of the study, the isolates were subcultured on antimicrobial agent-free medium to ensure viability and purity.* C. parapsilosis* ATCC 22019 and* C. krusei *ATCC 6258 (American Type Culture Collection, Manassas, VA) were used as quality controls and tested in each run of the experiments. All tests were performed in duplicate. MIC (minimum inhibitory concentration) values were determined visually as the lowest concentration of drug that caused significant growth diminution levels (≥ 50%). The MIC for AmB was defined as the lowest drug concentration at which growth was completely inhibited (100%).

The susceptibility values were interpreted taking into account the new species-specific clinical breakpoints suggested by the CLSI subcommittee for the most common species of* Candida* [9, 10]. Interpretive criteria for AmB and PSC have not been established, so based on reported data [11, 12] we selected a breakpoint of >1.0 mcg/mL to define isolates as AmB resistant and the species-specific breakpoints for VRC were used for PSC MIC values. The epidemiological cut-off value (ECV) of ≥1 *μ*g/mL was used to detect resistance in* C. glabrata* [9] (Table 1).

### 2.3. Statistical Analysis

The baseline characteristics of the patients included in the study were expressed by means, medians, and percentages. The incidence rate was calculated as the ratio between the number of new cases reported each year in the wards and the total number of patients hospitalized in the same facility. The association between qualitative variables was assessed by the chi-squared test. Through a univariate logistic model we tested the effect of each variable on the probability of death. Variables found to be significant in the univariate analysis were included in a multivariate logistic model. In all comparisons, a *P* < 0.05 was considered statistically significant. Data analysis was performed using the Statistical Package for the Social Sciences (SPSS) software 10 for Mac OS X (SPSS Inc., Chicago, IL, USA).

## 3. Results

During the 16 years of the study, 394 episodes of candidemia were identified, with an average incidence of 3.06/10 000 admissions (range 0.81–6.84). The median age of patients was 49.5 years (interquartile range, 9–67) and more candidemia occurred among males (65.7%). Most patients had one or more predisposing factors for candidemia: the use of broad-spectrum antibiotic treatment (for >15 days) was documented in 86.8% (342/394) of the cases, 340/394 (86.3%) patients had an intravascular catheter that only in 67% (228/340) of cases was removed, and 34.3% (135/394) of patients received total parenteral nutrition. At the time of candidemia diagnosis, 31.2% of patients (123/394) received antifungal prophylaxis with FLC (mean treatment 14.42 days, range 2–32). Out of 394* Candida* BSIs, 123 (31.2%) were diagnosed in the intensive care unit (ICU); 66 (16.8%) in the neonatal intensive care unit (NICU); 61 (15.5%) in general internal medicine; 57 (14.5%) in general surgery; 49 (12.4%) in paediatric oncohaematology; 23 (5.8%) in adult oncohaematology; and 15 (3.8%) in specialist surgery (neurosurgery and cardiac surgery).

Antifungal therapy was administered in 75.6% (298/394) of patients. Of these, liposomal AmB was the most frequently administered drug (182/298; 61.1%), whereas FLC was employed in 16.8% (50/298) of cases, CSP in 15.4% (46/298), and MCF in 3.4% (10/298). Combined therapy was administered in ten patients (10/298; 3.4%), six of whom received liposomal AmB plus 5-fluorocytosine and four of whom received VRC plus CSP.

**Figure 1:**
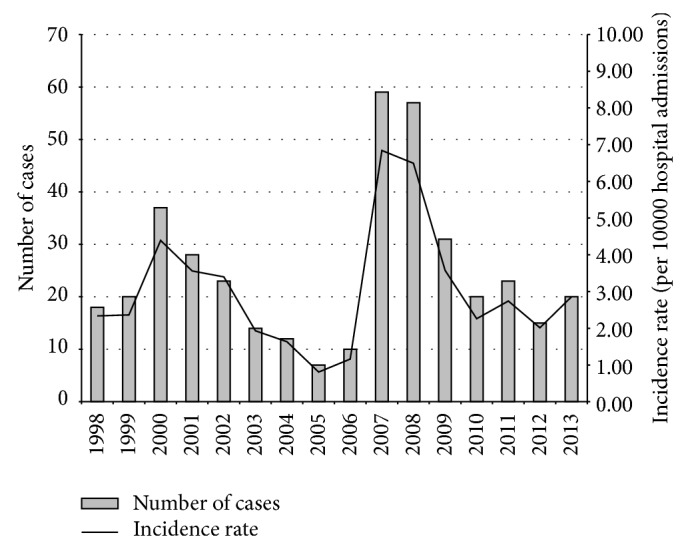
Patients with* Candida* BSI and incidence rate observed during a 16-year period.

During the years of observation, the* Candida* BSI incidence rate varied, increasing from 2.34 cases per 10 000 admissions in the year 1998 to 4.39 cases per 10 000 admissions in 2000, whereas in 2006 the incidence decreased significantly to 1.16 cases per 10 000 admissions (*P* < 0.0001). After a peak of 6.84 and 6.49 cases per 10 000 admissions in the years 2007 and 2008, respectively, the incidence rate decreased consistently and significantly to 2.86 cases per 10 000 admissions in the year 2013 (*P* < 0.0001) (Figure 1).

**Figure 2:**
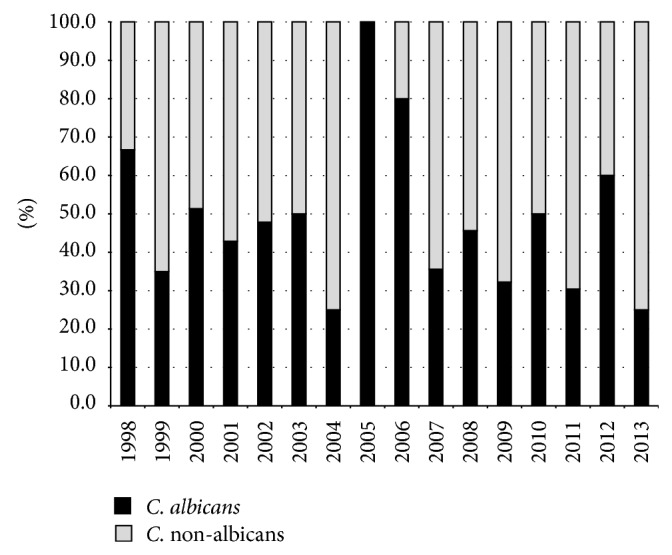
Temporal trend of* Candida albicans* and non*-albicans* during a 16-year period.

Considering the global distribution of species,* C. albicans* and non-*albicans* species caused 44.2% (174/394) and 55.8% (220/394) of the episodes, respectively. A slight variable drift of temporal trend of* Candida *spp. was observed during the 16 years of study, with a considerable percentage (75%) increase in non*-albicans *species in 2004 and 2013 (Figure 2). Among 220* non-albicans Candida *included* C. parapsilosis* (137; 62.2%),* C. glabrata* (22; 10%),* C. tropicalis* (19; 8.6%),* C. guilliermondii* (19; 8.6%), and* C. krusei* (11; 5%) whereas other species,* C. lusitaniae*,* C. norvegensis*,* C. inconspicua*,* C. famata*,* C. intermedia*,* C. zeylanoides, *and* C. pelliculosa,* were relatively rare (12; 5.4%).

**Figure 3:**
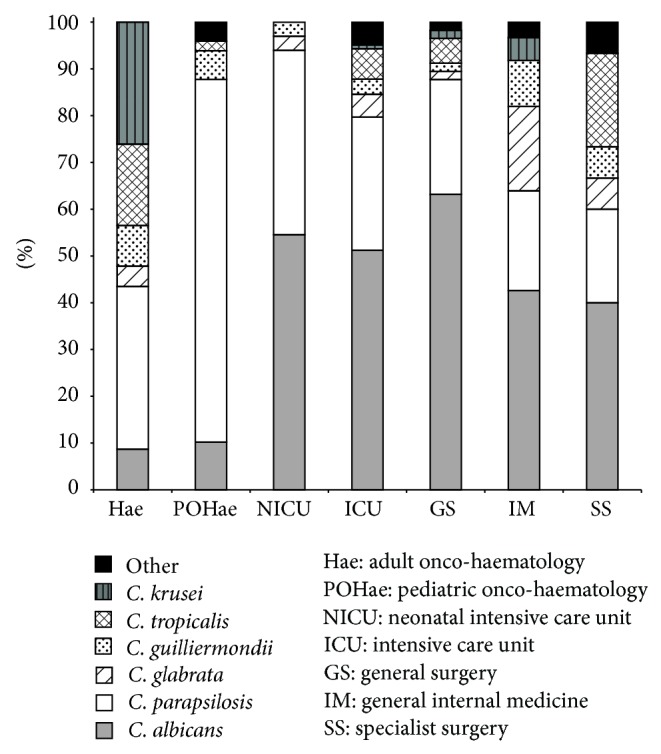
Distribution of* Candida* species according to hospital departments.

The dispersion of* C*.* albicans *and non-*albicans* isolates differed according to the patient population.* C. albicans* predominated in almost all departments, particularly in general surgery (63.2%), neonatal intensive care unit (54.4%), and general internal medicine (42.2%), whereas* C. parapsilosis* was the most frequently isolated species in adult and paediatric oncohaematology units (34.8% and 77.6%, resp.) (Figure 3).

*C. parapsilosis *showed a predilection toward younger patients of 1–17 years of age (39/137, 28.5%), whereas* C. albicans* was most frequently isolated in the age groups older than 40 years (113/174; 65.9%). Fourteen of 22 (63.6%)* C. glabrata* BSI cases were diagnosed in the age range 41–80 years.* C. krusei* was present in adults, in particular in the age groups of 41–80 years, whereas it was absent in children (Table 2).

Out of 123 patients (31.2%) receiving antifungal prophylaxis at the time of candidemia, 22 (17.9%) developed* C. albicans* BSI and the remaining 101 (82.1%) developed non*-albicans* infection, in particular by* C. parapsilosis* (61/101; 60.4%),* C. glabrata* (11/101; 10.9%),* C. tropicalis* (9/101; 8.9%),* C. krusei* (8/101; 7.9%), and* C. guilliermondii* (7/101; 6.9%). A significant dependence between prophylaxis and isolated* Candida* species resulted (*P* < 0.0001).

Overall, mortality occurred in 111 (28.2%) candidemic patients within 20 days from the onset of candidemia. Of those, 48 (43.2%) patients had BSI due to* C. albicans *and 63 (56.7%) to non-*albicans *species. We observed a significant dependence between death and hospital setting (*P* < 0.001) (Table 3); cases of death were higher in the ICU (58/123; 47.1%) and in specialist surgery (7/16; 43.7%).

A multivariate logistic model was built with the significant variables from a univariate analysis. The covariate “age,” “species,” and “department” were significant (age [*P* = 0.005], species [*P* = 0.012], and department [*P* = 0.001]). The Hosmer-Lemeshow Goodness-of-Fit of the model indicated that observed data matched expected data (chi-squared = 7.541; *P* = 0.480). The multivariate analysis indicated that* C. krusei* was associated with a statistically higher risk of death compared with both* C. albicans* (OR = 6.478; CL = 1.450, 28.935) and* C. parapsilosis* (OR = 7.909; CL = 1.731, 36.130).* C. glabrata* was also associated with a three times higher risk than* C. parapsilosis* (OR = 3.226; CL = 1.133, 9.185). In our analysis, age presented a risk of death associated with OR of 1.019 (CL = 1.006, 1.033) for an increase of 1 year of age.

Table 4 shows the results of* in vitro* susceptibility testing of the most commonly isolated species of* Candida *(*n* = 382). Most of the isolates were susceptible to the antifungal drugs tested. All azoles demonstrated good activity. In particular, only 5% (18/363) and 2.9% (10/341) of isolates were resistant to FLC and VRC, respectively. Resistance for both drugs was expressed mainly by* C. krusei *and* C. tropicalis*. PSC was inactive in 1.8% (6/341) of isolates. Similarly, 4.7% (18/382), 2.1% (8/382), and 1.3% (5/382) of isolates were resistant to CSP, AND, and MCF, respectively. Eight out of 174 (4.6%)* C. albicans *were resistant to CSP; 36.4% (4/11) of* C. krusei* and 21.1% (4/19) of* C. tropicalis* were resistant to CSP. Resistance to AND was mainly recognized in* C. parapsilosis*. All isolates were susceptible toAmB. When the annual geometric mean MIC values for the individual antifungals were plotted, only three drugs demonstrated an upward trend: FLC (*R* ^2^ = 0.27; exp *b* (0.38); *P* = 0.04), VCZ [*R* ^2^ = 0.443; exp *b* (0.031); *P* = 0.005), and PCZ (*R* ^2^ = 0.335; exp *b* (0.023); *P* = 0.355].

## 4. Discussion

Although considerable progress has been made in the management of patients with invasive fungal infections in the recent years,* Candida* BSIs are still widespread in hospital settings and closely associated with extended hospitalization and high healthcare costs. In the present study we evaluated the case trend of* Candida* BSIs during 16 years and although it was observed that the incidence per year was quite variable (range 0.81 to 6.84/10 000 admissions), the mean rate was 3.06/10 000 admissions and was in accordance with those reported from other centres. In fact, in Denmark, Arendrup et al. [14] observed an incidence of 4.1/10 000 admissions, in Israel Rennert et al. [15] reported 5/10 000 admissions, and in China Li et al. [16] reported 5.3/10 000 admissions; on the other side, some studies report higher incidence rates, as in Portugal, with 8.8/10 000 admissions [17], or in Brazil with 18.7/10 000 admissions [18]. This variability of fungal infection incidence rates among various geographical areas may reflect differences in healthcare practices among different countries in the populations studied, as well as the methodology adopted by different authors, or the systems for regular surveillance. In fact, in the present study we observed a maximum peak of incidence in the years 2007-2008 due to a rigorous active surveillance of invasive fungal infections that resulted in greater recruitment of cases [19–21].

Over the past 20 years, a shift toward non-*albicans Candida* species has been reported [1, 29], but the precise pattern of causative species varies across countries; hence epidemiological information available for one centre may not be applicable to others. Overall, in our study more than 50% of candidemia cases were due to non-*albicans Candida*. We observed a variable drift through 16 years and, in 2013, 75% of the cases were caused by non-*albicans Candida* species that were predominant in adult and paediatric patients with haematological malignancies (65/220; 29.5%).

According to other European reports [22–24], we found that* C. albicans* was the most frequent fungal species followed by* C. parapsilosis*, responsible for 62.3% of non-*albicans* episodes, whereas others report* C. glabrata* as the most common non-*albicans* species [1, 25]. The high profusion of* C. parapsilosis* could be explained by factors common in hospital settings, such as gastrointestinal colonization, or the affinity for intravascular devices and prosthetic materials. Additionally,* C. parapsilosis* has been known to colonize the hands of healthcare workers and is often responsible for nosocomial clusters, so its presence in hospital settings may be related to insufficient implementation of infection control measures. In our hospital during 1999,* C. parapsilosis* caused a BSI outbreak which affected children with haematological malignances, in which all* C. parapsilosis* isolates were genotypically identical between the strains isolated from the young patients and those isolated from the hands of one of the healthcare workers [26]. Although* C. parapsilosis* is less virulent than other species, its widespread dispersion and tendency to form biofilm on devices can interfere with antifungal therapy [27].

As in other studies [22, 28, 29], we observed a correlation of* Candida* species with patient age. BSIs due to* C. parapsilosis* showed a predilection toward younger patients, whereas* C. glabrata* and* C. tropicalis* were recovered from adult patients (>60 years old). The multivariate analysis showed that the species, independent of age and department, predicted the outcome of patients with candidemia. Certainly, the severity of underlying medical conditions also influences the mortality rate in these patients. Although resistance to antifungal agents is not common, the emergence of various non-*albicans Candida* species indicates the potential for development of antifungal resistance. For example, resistance to FLC is more common in non-*albicans Candida,* in part due to non-*albicans* species that are inherently resistant to antifungal, such as* C. krusei* [28, 30]. However, the uncontrolled use of echinocandin could promote the emergence of higher MIC values and promote the diffusion of* Candida* species such as* C. parapsilosis* or* C. guilliermondii* against which echinocandins have* in vitro* relatively less activity. In our study, the susceptibility rate to this drug class was very high during all periods of surveillance. Additionally, despite considerable use of FLC during these 16 years, the susceptibility rate to this agent continued to be very high, whereas the isolation of species historically less sensitive, such as* C. glabrata* and* C. krusei*, was low amounting to 22/394 and 11/394, respectively. This fact suggests that, apart from the use of FLC, other aspects may be important factors in the wide geographic variability of the species distribution, including demographic characteristics and use of antibiotics. However, it is important to note that the annual geometric mean MIC values of FLC demonstrated an upward trend during the 16 years, and the same tendency was observed for VRC and PSC. These data allow us to propose the possibility of cross-reaction among the azoles, considering that VRC and PSC have also been more recently introduced into clinical practice than FLC, underlying the need to monitor susceptibility trends. However, it is also important to consider that antifungal susceptibility testing has the limitation of correlating imperfectly with clinical outcomes. In fact, drug susceptibility does not guarantee clinical success because of the status of the host immune system and the persistence of infecting foci concurrent with illnesses influencing the outcome. Nevertheless, some isolates do not necessarily follow this general pattern. For this reason, knowing the susceptibility* in vitro* and monitoring the trend of MICs might facilitate the choice of appropriate therapy and provide guidance in the management of fungal infections. Additionally, the correct identification of fungal isolates is important in assessing epidemiology and in identifying geographic trends of the resistance profiles of most common* Candida* species.

## 5. Conclusion

This study emphasizes the importance of monitoring local epidemiologic data, considering the diversity of affected patient groups, knowing the antifungal susceptibility trend, and recognizing the important potential of such information to impact empiric antifungal therapy.

## Figures and Tables

**Table 1:** Species-specific breakpoints for antifungal agents for the most common species of *Candida* isolates.

Species	Antifungal agent	MIC (*µ*g/mL)			
		S	SDD	I	R
*C. albicans *	Amphotericin B	<1			≥1
	Fluconazole	≤2	4		≥8
	Voriconazole	≤0.12		0.25–0.5	≥1
	Posaconazole	≤0.12		0.25–0.5	≥1
	Anidulafungin	≤0.25		0.5	≥1
	Caspofungin	≤0.25		0.5	≥1
	Micafungin	≤0.25		0.5	≥1

*C. parapsilosis *	Amphotericin B	<1			≥1
	Fluconazole	≤2	4		≥8
	Voriconazole	≤0.12		0.25–0.5	≥1
	Posaconazole	≤0.12		0.25–0.5	≥1
	Anidulafungin	≤2		4	≥8
	Caspofungin	≤2		4	≥8
	Micafungin	≤2		4	≥8

*C. glabrata *	Amphotericin B	<1			≥1
	Fluconazole		≤32		≥64
	Voriconazole				≥1
	Posaconazole				≥1
	Anidulafungin	≤0.12		0.25	≥0.5
	Caspofungin	≤0.12		0.25	≥0.5
	Micafungin	≤0.06		0.12	≥0.25

*C. guilliermondii *	Amphotericin B	<1			≥1
	Fluconazole				
	Voriconazole				
	Posaconazole				
	Anidulafungin	≤2		4	≥8
	Caspofungin	≤2		4	≥8
	Micafungin	≤2		4	≥8

*C. tropicalis *	Amphotericin B	<1			≥1
	Fluconazole	≤2	4		≥8
	Voriconazole	≤0.12		0.25–0.5	≥1
	Posaconazole	≤0.12		0.25–0.5	≥1
	Anidulafungin	≤0.25		0.5	≥1
	Caspofungin	≤0.25		0.5	≥1
	Micafungin	≤0.25		0.5	≥1

*C. krusei *	Amphotericin B	<1			≥1
	Fluconazole	—	—		—
	Voriconazole	≤0.5		1	≥2
	Posaconazole	≤0.5		1	≥2
	Anidulafungin	≤0.25		0.5	≥1
	Caspofungin	≤0.25		0.5	≥1
	Micafungin	≤0.25		0.5	≥1

Data compiled from [9–12].

S, susceptible; SDD, susceptible-dose-dependent; I, intermediate; R, resistant.

All *C. krusei* isolates were considered intrinsically resistant to fluconazole [9].

**Table 2:** Distribution of *Candida* spp. isolates for different age groups.

*Candida* species	Patients age groups													
	<1		1–17		18–40		41–60		61–80		>80		Total	
	*n*	%	*n*	%	*n*	%	*n*	%	*n*	%	*n*	%	*n*	%
*C. albicans *	33	19.0	12	6.9	16	9.2	49	28.2	53	30.5	11	6.3	174	100
*C. parapsilosis *	25	18.2	39	28.5	13	9.5	27	19.7	25	18.2	8	5.8	137	100
*C. glabrata *	2	9.1	0	0.0	3	13.6	5	22.7	9	40.9	3	13.6	22	100
*C. guilliermondii *	2	10.5	5	26.3	1	5.3	5	26.3	3	15.8	3	15.8	19	100
*C. tropicalis *	0	0.0	2	10.5	1	5.3	7	36.8	9	47.4	0	0.0	19	100
*C. krusei *	0	0.0	1	9.1	1	9.1	5	45.5	4	36.4	0	0.0	11	100
Other *Candida *spp.^∗^	0	0.0	2	16.7	4	33.3	2	16.7	2	16.7	2	16.7	12	100

Total	62	15.7	61	15.5	39	9.9	100	25.4	105	26.6	27	6.9	394	100

^∗^ *Candida* spp.: *C. lusitaniae, C. norvegensis, C. inconspicua, C. famata, C. intermedia, C. zeylanoides*, and *C. pelliculosa.*

**Table 3:** Risk analysis of death with a univariate logistic mode.

	Death *N* = 111	Living *N* = 283	*p* (Wald test)
Age, years (median, I and III quartile)	58.0 (44.0–74.0)	42.0 (8.0–64.0)	**<0.0001** ^∗^
Gender, *n* (%)			
Male	72 (64.9)	187 (66.1)	0.819
Female	39 (35.1)	96 (33.9)	
Catheter, *n*. (%)			
Yes	99 (89.2)	241 (85.2)	0.128
No	12 (10.8)	42 (14.8)	
Antibiotic therapy, *n*. (%)			
Yes	97 (87.4)	245 (86.6)	0.830
No	14 (12.6)	38 (13.4)	
Antifungal prophylaxis, *n*. (%)			
Yes	36 (32.4)	87 (30.7)	0.745
No	75 (67.6)	196 (69.3)	
*Candida *species, *n*. (%)			
*C. albicans *	48 (43.2)	126 (44.5)	**0.001**
*C. parapsilosis *	24 (21.6)	113 (9.9)	
*C. glabrata *	11 (9.9)	11 (3.9)	
*C. guilliermondii *	7 (6.3)	12 (4.2)	
*C. tropicalis *	8 (7.2)	11 (3.9)	
*C. krusei *	6 (5.4)	5 (1.8)	
Other	7 (6.3)	5 (1.8)	
Department, *n*. (%)			
Adult oncohaematology	5 (4.5)	18 (6.4)	**<0.001** ^**∗∗**^
Pediatric oncohaematology	—	49 (17.3)	
NICU	12 (10.8)	54 (19.1)	
ICU	58 (52.2)	65 (23)	
General surgery	12 (10.8)	45 (15.9)	
General internal medicine	17 (15.3)	43 (15.2)	
Specialist surgery	7 (6.3)	8 (2.8)	

NICU: neonatal intensive care unit.

ICU: intensive care unit.

^∗^Wilcoxon test.

^∗∗^The pediatric haematology patients were excluded from analysis because none of the candidemic patients died.

**Table 4:** *In vitro* susceptibility to the antifungal agents against 394 *Candida* isolates.

Species (number of isolates)	Antifungal agent	MIC (*µ*g/mL)		
		Range	50%	90%
*C. albicans *(174)	Amphotericin B	0.06–1	0.50	1
	Fluconazole	0.06–2	0.25	1
	Voriconazole	≤0.008–0.5	0.008	0.25
	Posaconazole	≤0.008–0.5	0.03	0.25
	Anidulafungin	≤0.008–1	0.06	0.25
	Caspofungin	≤0.008–1	0.25	0.5
	Micafungin	≤0.008–0.5	0.03	0.06

*C. parapsilosis *(137)	Amphotericin B	0.06–1	0.25	0.5
	Fluconazole	0.06–8	0.5	1
	Voriconazole	≤0.008–1	0.015	0.06
	Posaconazole	≤0.008–2	0.125	0.25
	Anidulafungin	≤0.008–32	1	2
	Caspofungin	0.125–8	2	2
	Micafungin	≤0.008–32	1	2

*C. glabrata* (22)	Amphotericin B	0.25–1	0.5	1
	Fluconazole	2–32	8	16
	Voriconazole	0.03–2	0.25	1
	Posaconazole	0.25–4	0.25	4
	Anidulafungin	≤0.008–1	0.25	0.25
	Caspofungin	≤0.008–4	0.06	0.5
	Micafungin	≤0.008–1	0.06	0.5

*C. guilliermondii* (19)	Amphotericin B	0.125–1	0.25	1
	Fluconazole	0.5–4	4	4
	Voriconazole	≤0.008–1	0.06	0.5
	Posaconazole	0.125–1	0.5	1
	Anidulafungin	1–4	1	2
	Caspofungin	1–4	2	4
	Micafungin	0.5–2	1	2

*C. tropicalis* (19)	Amphotericin B	0.06–1	0.5	1
	Fluconazole	0.125–64	0.5	64
	Voriconazole	0.008–16	0.06	8
	Posaconazole	0.015–2	0.125	0.5
	Anidulafungin	0.03–0.25	0.25	0.25
	Caspofungin	≤0.008–2	0.06	1
	Micafungin	0.0015–1	0.25	0.5

*C. krusei *(11)	Amphotericin B	0.5–1	1	1
	Fluconazole	8–64	32	32
	Voriconazole	0.125–8	0.25	1
	Posaconazole	0.25–2	0.5	2
	Anidulafungin	≤0.008–0.5	0.25	0.5
	Caspofungin	0.06–2	0.25	1
	Micafungin	0.06–0.5	0.25	0.5
